# Breast cancer response to neoadjuvant chemotherapy: predictive markers and relation with outcome

**DOI:** 10.1038/sj.bjc.6600749

**Published:** 2003-02-10

**Authors:** I F Faneyte, J G Schrama, J L Peterse, P L Remijnse, S Rodenhuis, M J van de Vijver

**Affiliations:** 1 Department of Pathology, The Netherlands Cancer Institute, Plesmanlaan 121, 1066 CX Amsterdam, The Netherlands; 2 Division of Experimental Therapy, The Netherlands Cancer Institute, Plesmanlaan 121, 1066 CX Amsterdam, The Netherlands; 3 Divsion of Medical Oncology, The Netherlands Cancer Institute, Plesmanlaan 121, 1066 CX Amsterdam, The Netherlands

**Keywords:** breast cancer, neoadjuvant chemotherapy, tumour response, predictive markers

## Abstract

The aim of this study was to provide a better insight into breast cancer response to chemotherapy. Chemotherapy improves outcome in breast cancer patients. The effect of cytotoxic treatment cannot be predicted for individual patients. Therefore, the identification of tumour characteristics associated with tumour response and outcome is of great clinical interest. We studied 97 patients, who received anthracycline-based neoadjuvant chemotherapy. Tumour samples were taken prior to and after chemotherapy. We quantified the response to chemotherapy clinically and pathologically and determined histological and molecular tumour characteristics. We assessed changes in the expression of Bcl-2, ER, P53 HER2 and Ki-67. Association with response and outcome was tested for all parameters. The experimental results showed 15 clinical (17%) and three (3%) pathological complete remissions. There were 18 (20%) clinical *vs* 29 (33%) pathological nonresponders. The expression of most markers was similar before and after chemotherapy. Only Ki-67 was significantly decreased after chemotherapy. Factors correlated with response were: large tumour size, ER negativity, high Ki-67 count and positive P53 status. Tumour response and marker expression did not predict disease-free or overall survival. In conclusion, clinical and pathological response assessments are poorly associated. Proliferation decreases significantly after chemotherapy. ER negativity and a high proliferation index are associated with better response. HER2 status does not predict response, and outcome is not related to tumour response.

The addition of adjuvant chemotherapy to standard breast cancer therapy has been shown to improve outcome substantially (Early Breast Cancer Trialists' Collaborative Group, 1998). There are several arguments for applying chemotherapy in a so-called neoadjuvant treatment setting, prior to surgery. First, by downstaging the tumour, less extensive resections are needed and breast conservation becomes increasingly feasible (Makris *et al*, 1998; Mauriac *et al*, 1999). Second, micrometastases that may be present are thus treated at the earliest possible moment. This could prevent changes in metastatic cells, associated with a worse prognosis: the acceleration of growth upon resection of the primary tumour (Gunduz *et al*, 1979; Fisher *et al*, 1983) and the development of drug-resistant subclones (Goldie and Coldman, 1979). A third advantage of neoadjuvant chemotherapy is that it enables the monitoring of treatment efficacy and makes it possible to identify markers of response to chemotherapy.

Most published data on the effect of cytotoxic treatment in breast cancer have been obtained by comparing the survival of large groups of patients with or without chemotherapy. A more direct approach is to measure the primary tumours of individual patients before and after treatment. Assuming that the response – that is, decrease of tumour volume – is representative of the sensitivity of all tumour cells, this could yield a prognostic tool: a better response could be associated with an improved outcome.

It has not been demonstrated in what way the effects of chemotherapy exposure should be measured to obtain a valid parameter of the response. Whether the response of breast cancer to chemotherapy actually is a prognostic factor is also not an established fact. There are many studies investigating the prognostic factors in breast cancer, such as the expression of HER2 (reviewed in Hamilton and Piccart, 2000), P53 (reviewed in Bergh, 1999) and Bcl-2 (reviewed in Daidone *et al*, 1999). In addition, there are an increasing number of studies investigating factors that can predict response to chemotherapy. Histopathological changes of breast tumours as a result of chemotherapy exposure have been described in a limited number of papers (Kennedy *et al*, 1990; Aktepe *et al*, 1996; Miller *et al*, 1997; Gajdos *et al*, 2002).

In this study, we have made a comparison of the same set of tumours before and after exposure to chemotherapy, describing changes in three aspects: size, histopathologic features, and expression of molecular markers. We have correlated these factors with overall (OS) and disease-free survival (DFS) of the patients.

The main aim of this study was to provide a better insight into breast cancer response to chemotherapy by identifying factors that can predict response of the primary tumour to neoadjuvant anthracycline-based chemotherapy and by assessing the association between tumour response and outcome.

## MATERIALS AND METHODS

### Patients

All patients (*n*=97) presented with breast cancer with extensive axillary lymph node involvement. The diagnosis of breast cancer was made by clinical, radiological and cytological assessment in all patients. For those patients with inadequate cytological aspirates, histological confirmation of the diagnosis was obtained with primary tumour biopsy. Patients were entered in a single-institution, randomised trial, studying the effects of high-dose chemotherapy and haematopoietic progenitor-cell support transplantation. The results of this study were published earlier (Rodenhuis *et al*, 1998). In short, the eligibility criteria of this study were: age under 60 years, level III involvement of axillary lymph nodes (detected with an infraclavicular histological lymph node biopsy that was performed in all patients and had to be tumour-positive to be entered in the trial), absence of distant metastases and locally resectable disease. Patients received three courses of cyclophosphamide (500 mg m^−2^), epirubicin (120 mg m^−2^) and 5-fluorouracil (500 mg m^−2^) (FE_120_C) once every 3 weeks as up-front neoadjuvant therapy. All but one patient finished the up-front chemotherapy, and 94% did so within the planned time frame. Following up-front treatment, patients underwent definitive surgery, consisting of either a mastectomy (*n*=81) or breast-conserving surgery (*n*=16) and axillary lymph node dissection. After surgery, patients were randomly assigned to receive conventional chemotherapy alone (one more cycle of FE_120_C) or FE_120_C followed by an additional high-dose course (cyclophosphamide 6 g m^−2^, thiotepa 480 mg m^−2^, carboplatin 1600 mg m^−2^) and peripheral-blood progenitor-cell support after the fourth FE_120_C course. All patients received radiation therapy and 2 years of tamoxifen upon finishing the respective chemo-therapy regimens. Written informed consent was obtained from all patients before enrolment in the study. Patients were again asked for their permission to participate after neoadjuvant chemotherapy and surgery and before randomisation.

### Histology

The assessable tumours were graded and classified from haematoxylin and eosin (H&E)-stained sections of the surgical resection specimen obtained after chemotherapy treatment. In cases of (nearly) complete primary tumour remission, the residual axillary node metastases were used to grade and type the tumour. Histological classification was done according to the WHO criteria. Grading was done according to the criteria described by Elston and Ellis (1991).

### Response to FE_120_C – clinical assessment

Clinical response was categorised according to the UICC criteria (Hayward *et al*, 1977). A clinical complete remission (cCR) was defined as the disappearance of all palpable tumour deposits. Clinical response was scored as partial remission (cPR) if the reduction of tumour volume exceeded 50%. Tumour reduction less than 50% or increase of volume up to 25% was scored as stable disease (cSD). An increase of more than 25% was designated as progressive disease.

### Response to FE_120_C – pathological assessment

Histopathological evidence of response was scored in H&E sections of the surgical specimen. Only invasive carcinomas – of any subtype – were studied, and the presence or absence of *in situ* carcinoma was disregarded. The words *tumour* and *response* are therefore consistently used when referring to invasive carcinomas. Quantitatively we scored if and where tumour deposits remained after up-front chemotherapy: at the site of the primary tumour (diameter) and in the axilla (number of positive nodes *vs* total number of retrieved nodes). Qualitatively we scored morphologic alterations of tumour cells and surrounding tissue. From an earlier pilot study, comparing the first 17 patients from this trial group to 17 randomly selected untreated breast carcinoma control cases (unpublished data of JLP and PLR), we defined two characteristics associated with chemotherapy exposure: *fibrosis* and *cytonuclear changes*. Fibrosis, resembling scar tissue, appeared as hyaline-rich, cell-deprived, desmoplastic stroma and was only scored thus if located at the peripheral edges of the tumour; central hyalinisation is frequently seen in untreated tumours. Regressive cytonuclear alterations were scored if marked anisocytosis, vacuolisation and increase of nuclear size was seen in residual tumour cells. The few publications addressing the subject of breast cancer morphology after chemotherapy essentially describe the same pattern of changes that we found (Kennedy *et al*, 1990; Aktepe *et al*, 1996; Miller *et al*, 1997; Gajdos *et al*, 2002).

A pathological complete remission (pCR) was defined if no residual carcinoma was seen macroscopically and microscopically. If minimal tumour residues were found in the breast or the axilla, this was scored as microscopic residual disease (pMRD). Cases with histopathological characteristics of chemotherapy exposure – as described above – but a macroscopic residual tumour mass were categorised as histopathological changes in a macroscopic residual tumour mass (pHRTM). All cases, in which no qualitative or quantitative histopathologic evidence of tumour response was seen, were scored as no response (pNR).

### Immunohistochemistry

For 56 patients, material of the tumour obtained prior to chemotherapy was available for immunohistochemistry (IHC): a histological biopsy of the primary tumour (*n*=8) or a histological biopsy of the infraclavicular lymph node (*n*=48). For the other patients what was left of the prechemotherapy biopsy material after diagnostic work-up was too little to process with IHC. For the postchemotherapy material the problem of limited quantity was also met, however to a lesser extent. Of 86 patients the surgical resection specimen obtained after neoadjuvant chemotherapy was still available for IHC.

Formalin-fixed paraffin-embedded tissue samples were stained with antibodies against HER2 (3B5; 1 : 10 000), P53 (DO7; 1 : 8000; mouse IgG2b; DAKO, Denmark), oestrogen receptor (ER) (1D5; 1 : 150; mouse IgG1; DAKO, Denmark), Ki-67 (MIB-1; 1 : 1000; mouse IgG1; Immunotech, France) and Bcl-2 (clone 124; 1 : 100; mouse IgG1; DAKO, Denmark). Sections of 5 *μ*m were deparaffinised in xylol and alcohol, and endogenous peroxidase was quenched in methanol-peroxide (3%; 20′). Slides were pretreated (antigen retrieval) in citrate buffer (0.01 M citric acid, in distilled water (pH 6.0); 15′ at 100°C), blocked with normal goat serum (5%; 30′) and then incubated with the primary antibody, overnight at 4°C. Binding of the monoclonal antibody was detected with biotin-labelled goat anti-mouse IgG (1 : 200; 30′; DAKO, Denmark) and horseradish peroxidase-labelled avidin – biotin complex (1 : 100; 30′; DAKO, Denmark). Bound peroxidase was developed with 3,3′-diaminobenzidine tetrahydrochloride (Sigma, USA) and 0.02% H_2_O_2_ in PBS. All reagents were diluted in a 1% bovine albumin solution in PBS. Anti-mouse IgG solution was admixed with 10% normal human serum to prevent nonspecific binding. Replacement of the primary antibody with 1% bovine albumin solution in PBS served as negative control.

IHC results of Bcl-2, ER and P53 were scored semiquantitatively on a six-point scale for the percentage of positively staining tumour cells (0; 1<10%; 2=10–25%; 3=25–50%; 4=50–75%; 5=75–100%). Staining patterns recognised as positive were circumferential membrane-bound staining (HER2), nuclear staining (ER, Ki-67, P53) or cytoplasm staining (Bcl-2). HER2 was scored with the system that has recently come to use for clinical testing (0; 1+=>10% cells weakly positive; 2+=moderate homogeneous staining; 3+=strong homogeneous staining). Ki-67 (the proliferating fraction) was scored quantitatively by counting the number of positive tumour cells in a total of 200 per slide.

### Survival

We determined both OS and DFS from the start of neoadjuvant treatment. End points were death for OS and death or any recurrence of breast cancer for DFS. The original trial design had two randomly assigned treatment protocols: a high-dose chemo-therapy arm and a standard chemotherapy arm, as described above. Survival comparisons between groups have been published (Rodenhuis *et al*, 1998). The outcome was reanalysed after 7 years of follow-up and was still identical for both treatment groups (Schrama *et al*, 2002). For the analysis of predictive factors, we have therefore used the total patient population regardless of the treatment arm they were assigned to.

### Analysis of results

All data were analysed with SPSS 10.0.7 for Windows. For correlation between expression levels of markers and response parameters, we used the two-sided Spearman's test for nonparametric correlation. For crosstabulation, markers were categorised dichotomously as *positive* or *negative.* The cut-off for Bcl-2, ER and P53 was at 10% or more tumour cells staining (score >1). For HER2, 2+ and 3+ were regarded as *positive*. Ki-67 was dichotomised in *low* (less than 40 staining nuclei out of 200) *vs high* (40 or more staining nuclei out of 200). Differences of expression levels between paired samples (pre- *vs* post-chemotherapy) scored on a six-point scale were assessed with Wilcoxon's signed ranks test. Significance of changes in dichotomised status was tested with McNemar's test. Response was dichotomised depending on the way in which it was assessed: clinically as *cCR vs non-cCR* and pathologically as *any response (pCR, pMRD or pHRTM) vs no response (pNR)*. Association between dichotomised scores of marker expression and tumour response was tested with the Fisher exact test. Analyses of survival differences between the patient subgroups were carried out with the log-rank test. Response scores and marker expression were also tested for association with outcome in a Cox regression model.

## RESULTS

In tumour samples taken from 97 breast cancer patients with extensive lymph node involvement, who were preoperatively treated with three courses of FE_120_C, response to neoadjuvant chemotherapy was measured. The association with response and survival of histopathological features and of a panel of markers was tested. In addition, we compared marker expression before and after chemotherapy in the same tumour, and response to chemotherapy was tested as a possible predictor of outcome.

### Response to FE_120_C

Clinical and pathological response data were available for 89 patients.

Upon three cycles of FE_120_C, 15 patients (17%) had a clinical complete remission (cCR), 56 had a partial remission (cPR) (63%) and 18 had stable disease (SD) (20%). Progressive disease was not observed.

Histopathologic signs of response were seen in 60 patients (67%). Three patients had a pCR (3%) and only pMRD was found in 11 cases (12%). In 46 cases (52%) pHRTM was found. In all, 29 patients (33%) had pNR. Including three patients with a pCR, 15 patients (17%) had no residual tumour in the breast after neoadjuvant treatment and four (4%) had a tumour-negative axilla. The mean tumour diameter measured in the surgical sample after neoadjuvant chemotherapy was 2.6 cm (range 0–10 cm).

Of the 15 patients with cCR, two (13%) also had a pCR and four (27%) had pMRD. In nine cCR patients (60%) only pHRTM (*n*=7) or pNR (*n*=2) was observed. Of three patients with a pCR, two were also classified as cCR and one was clinically scored as cPR. In all, 10 out of 29 pNR cases were clinically scored as cSD (34%), 17 were scored as cPR (59%) and two as cCR (7%). These data are summarized in Table 1

**Table 1:** (A) Histopathological findings in patients with a clinical complete remission and (B) clinical findings in patients with no histopathological signs of response

**(A) Pathological response**	**cCR (*n*=15)**	**(B) clinical response**	**pNR (*n*=29)**
pCR	2	CCR	2
pMRD	4	cPR	17
pHRTM	7		
pNR	2	cSD	10
Any pathological sign of response	*13* (*87*%)	Any clinical sign of response	*19* (*66*%)

Histopathological examination: pCR=pathological complete remission; pMRD=minimal residual disease; pHRTM=histopathological changes in macroscopic residual tumour mass; pNR=no histopathological signs of response.

Clinical examination: cCR=clinical complete remission; cPR=partial response; cSD=stable disease.

.

### Integrated clinical and histopathological definition of response

The correlation between clinical and pathological response was not very strong, but highly significant (*ρ*=0.34; *P*=0.001). Aiming to take into account as many parameters as possible to define the degree of tumour response, we reclassified the patients into seven categories integrating both clinical and histopathological response measurement:

- Pathological complete remission (pCR; *n*=*3*).
- Microscopic residual disease and clinical complete remission (pMRD and cCR; *n*=4).
- Microscopic residual disease, but no clinical complete remission (pMRD and cPR/cSD; *n*=7).
- Histopathological changes in a macroscopic residual tumour mass and a clinical complete or partial remission (pHRTM and cCR/cPR; *n*=39).
- A clinical complete or partial remission but no pathological response (pNR and cCR/cPR; *n*=19).
- Histopathological changes in a macroscopic residual tumour mass and clinically stable disease (pHRTM and cSD; *n*=7).
- No pathological response and clinically stable disease (pNR and cSD; *n*=10).

### Characteristics of patients and tumours

The mean age of patients at diagnosis was 45 years. A histological classification could be made for 76 tumours: 49 were classified as invasive ductal carcinoma (IDC) (64.5%), 11 as invasive lobular carcinoma (ILC) (14.5%) and 16 as other types (21%), including mixed invasive patterns. Malignancy grading was possible for 73 tumours: 25 (34%) were grade I, 30 were grade II (41%) and 18 (25%) were grade III.

### Expression of markers

IHC was performed with sections from as many pre- and postchemotherapy histological biopsy samples as were available. We had 56 prechemotherapy samples:

- primary tumour (*n*=8),
- infraclavicular lymph node (*n*=48).

We had 86 samples after chemotherapy, all from the primary tumour surgery specimen. Of 50 patients, material from before and after the up-front FE_120_C courses was available. As a result of technical problems (sections were not sufficiently adherent to glass slides), less than 50 sample pairs were stained with assessable results for Bcl-2 and ER. These data are summarized in Table 2

**Table 2:** IHC results of five markers in sample pairs taken prior to and after FE_120_C neoadjuvant treatment

		**Unchanged**				
	***N***	**Pos**	**Neg**	**Pos→Neg**	**Neg→Pos**	***P*-value**
Bcl-2	43	16	8	9	10	NS
ER	49	25	10	7	7	NS
HER2	50	12	35	2	1	NS
P53	50	15	24	5	6	NS
		High	Low	High→Low	Low→High	*P*-value
						
Ki-67	50	5	26	15	4	*0.019*

Unchanged=status remains unchanged after chemotherapy exposure. Pos=marker status is positive (>10% staining cells). Neg=marker status is negative (0–10% staining cells). Pos→Neg (v.v.)=marker status changed from positive to negative after chemotherapy exposure (v.v.). *P*-value=significance of changes in marker expression after chemotherapy was tested with McNemar's test.

.

Bcl-2 was assessed in 43 sample pairs pre- and postchemotherapy and Bcl-2 status remained the same in 24 (56%). In 10 patients, Bcl-2 was negative in the prechemotherapy biopsy and positive in the postchemotherapy resection specimen. In nine cases, Bcl-2 was positive before chemotherapy and negative after. The difference in Bcl-2 expression level before and after chemotherapy exposure was not statistically significant.

ER was assessed in 49 pre- and postchemotherapy sample pairs and remained the same in 35 (71%). In seven patients, ER was negative in the prechemotherapy biopsy and positive in the postchemotherapy resection specimen. In seven cases, ER was positive before chemotherapy and negative after. The difference in ER expression level before and after chemotherapy exposure was not statistically significant.

HER2 was assessed in 50 pre- and postchemotherapy sample pairs and remained the same in 47 (94%). In one patient, HER2 was negative in the prechemotherapy biopsy and positive in the postchemotherapy resection specimen. In two cases, HER2 was positive before chemotherapy and negative after. The difference in HER2 expression level before and after chemotherapy exposure was not statistically significant.

P53 was assessed in 50 pre- and postchemotherapy sample pairs and remained the same in 39 (78%). In six patients, P53 was negative in the prechemotherapy biopsy and positive in the postchemotherapy resection specimen. In five cases, P53 was positive before chemotherapy and negative after. The difference in P53 expression level before and after chemotherapy exposure was not statistically significant.

Ki-67 was assessed in 50 pre- and postchemotherapy sample pairs and remained the same in 31 (62%). Ki-67 count was high in 20 patients (40%) before treatment and in nine (18%) after. In four patients, Ki-67 was low in the prechemotherapy biopsy and high in the postchemotherapy resection specimen. In 15 cases, Ki-67 was high before chemotherapy and low after. The mean proliferating fraction was 38 per 200 before and 22 per 200 after chemotherapy exposure as counted in 50 pairs of tumour samples. This difference was statistically significant (*P*=0.000053; two-sided t-test for paired samples).

### Correlating tumour characteristics with response to FE_120_C 

Prechemotherapy patient and tumour characteristics were tested for correlation with integrated histopathological and clinical tumour response. The tumour diameter at diagnosis was correlated inversely with response (*ρ*=−0.27; *P*=0.017), as was ER expression (*ρ*=−0.40; *P*=0.003). P53 expression was positively correlated (*ρ*=0.28; *P*=0.046) with response.

The integrated response score was dichotomised as I–III or IV–VII and crosstabulated *vs* all markers as *positive* or *negative.* Of 19 ER-negative tumours; six (32%) were in the best-response group *vs* two (6%) out of 34 ER-positive tumours (*P*=0.02; Fisher's exact). P53-positive tumours were more frequently in the best-response group (6 of 22; 27%) than P53-negative tumours (two of 31; 6%) (*P*=0.05). Tumours with a high Ki-67 count (*P*=0.02) were more likely to have a better response (seven of 24; 29%) than low Ki-67 tumours (one of 29; 3%).

Bcl-2 and HER2 expression were not correlated with response to FE_120_C. Table 3

**Table 3:** Significance levels (Fisher's exact test; *P*-values) of association between IHC-detected marker expression (sample before FE_120_C) and response by clinical, pathological and integrated assessment, all dichotomised and thus crosstabulated

	**Clinical cCR *vs* non-cCR**	**Pathological Any *vs* none**	**Combination I–III *vs* IV–VII**
ER (*n*=53)	0.73	0.04*	0.02*
Positive 34		Any 18; None 16	I–III 2; IV–VII 32
Negative 19		Any 16; None 3	I–III 6; IV–VII 13
			
P53 (*n*=53)	0.07	0.04*	0.05*
Positive 22		Any 18; None 4	I–III 6; IV–VII 16
Negative 31		Any 16; None 15	I–III 2; IV–VII 29
			
Ki-67 (*n*=53)	0.03*	0.78	0.02*
High 24	CR 8; non-CR 16		I–III 7; IV–VII 17
Low 29	CR 2; non-CR 27		I–III 1; IV–VII 28
			
Bcl-2 (*n*=49)	0.24	1.0	1.0
			
HER2 (*n*=53)	0.25	0.76	1.0

* Association is statistically significant at the 0.05 level.

gives significance of associations between marker expression and all three response scores.

In a binary logistic regression model comprising all tested markers as covariates and the integrated response score as a dependent variable (all dichotomised as indicated), only the Ki-67 score was independently associated with response (*P*=0.04).

### Response to FE_120_C and survival

There was no association between response of the primary tumour and OS. No statistically significant differences of OS between the patient subgroups were found with either definition of response.

Log-rank analysis (Table 4

**Table 4:** (A) OS (*P*=0.1; *log-rank statistics*) and (B) DFS (*P*=0.04; *log-rank statistics*) for patient subgroups, by integrated pathological and clinical tumour response

	**Integrated clinical and pathological response**	***N***	**No. of patients^a^**	**5-years OS (%)**	**Median OS in years (95% CI)**
A, OS					
I.	pCR	3	2	33	1.5 (0.9–2.1)
II.	pMRD and cCR	4	3	25	3.4 (0.6–6.2)
III.	pMRD and cPR/cSD	7	1	100	7.3 (7.1–7.6)
IV.	pHRTM and cCR/cPR	39	20	61	6.5 (4.7–8.2)
V.	pNR and cCR/cPR	19	10	58	5.6 (ND)
VI.	pHRTM and cSD	7	5	43	3.1 (0–6.6)
VII.	pNR and cSD	10	5	47	4.3 (ND)
	**Integrated clinical and pathological response**	***N***	**No. of patients^b^**	**5-years OS (%)**	**Median OS in years (95% CI)**
B, DFS					
I.	pCR	3	3	33	1.3 (0.7–2.0)
II.	pMRD and cCR	4	3	25	1.7 (0.3–3.1)
III.	pMRD and cPR/cSD	7	1	86	ND (ND)
IV.	pHRTM and cCR/cPR	39	25	44	4.3 (3.3–5.3)
V.	pNR and cCR/cPR	19	11	47	3.7 (0–7.6)
VI.	pHRTM and cSD	7	6	14	3.0 (0–9.0)
VII.	pNR and cSD	10	5	50	3.9 (ND)

a Number of patients who died during follow-up.

b Number of patients with an event during follow-up (death or recurrent disease).

ND=could not be determined.

) did show significant association of DFS and response, but only if the integrated response score was used (*P*=0.04). The integrated response score did, however, not predict a trend of increased DFS with increasing response (*P*=0.9; Cox regression): group III patients (pMRD and cPR/cSD) had the longest DFS, whereas group I patients (pCR) relapsed relatively fast. Patients with cCR (*n*=15) had a 5-year OS of 60%. This was not significantly different from non-cCR patients (*n*=82) who had a 5-year OS of 57% (*P*=0.44). The 5-year DFS rates (cCR=60%; non-cCR=41%) were also not significantly different (*P*=0.38).

The outcome in patients with any histopathological sign of response (pCR, pMRD or pHRTM; *n*=60) was equal to that in patients with no signs of response (pNR; *n*=29) in terms of 5-year OS (59 and 54%, respectively; *P*=0.97) and 5-year DFS (43 and 48%, respectively; *P*=0.59).

Unexpectedly, patients with pCR (*n*=3) had a relatively poor outcome as two patients relapsed and died within 18 months and the third was alive with a recurrence at the time of last follow-up, 5.4 years after treatment. There was no association between the expression of any of the IHC-determined markers and OS or DFS.

## DISCUSSION

In a well-defined series of breast cancer patients (Rodenhuis *et al*, 1998), we have studied chemotherapy-induced changes of tumour phenotype. The overall aim of the study was to provide a better insight into breast cancer response to chemotherapy.

To quantify tumour response, we used both the palpated tumour sizes before and after chemotherapy and the residual tumour volume in the surgical specimen. Although clinical response is clearly correlated to findings at histopathological examination, it does not appear to be an adequate parameter to use for assessment of therapeutic success. It has been demonstrated before that concordance between clinical and pathological findings in breast cancer patients treated with primary chemotherapy is moderate at best (Smith *et al*, 1995; Chollet *et al*, 1997). Our results show that measurement by palpation overestimates the number of complete remissions and underestimates the number of nonresponders, as compared to histological assessment of tumour regression (Table 1). We therefore used an additional scale for tumour response, integrating both reduction in tumour size and histopathologic changes.

We show that the expression of most of the tested markers does not change after FE_120_C treatment (Table 2). Schneider *et al* (2000) studied the expression of several markers in 48 pre- and postchemotherapy breast carcinoma samples and reached the same conclusion. In earlier publications, histological tumour parameters were also determined before and after chemotherapy, and were also found to remain largely unchanged (Frierson Jr and Fechner, 1994; Sharkey *et al*, 1996). This implies that these markers can be determined reliably in the prechemotherapy biopsy as well as in the postchemotherapy surgical specimen. With the increasing use of preoperative chemotherapy and with the growing insight into molecular markers, this is an important finding.

The only marker that was expressed differently in pre- *vs* postchemotherapy samples was Ki-67, representing the proliferating fraction of the tumour. This finding is in line with studies showing that doxorubicin treatment results in a significant decrease of the mitotic activity (Honkoop *et al*, 1997) and of the number of Ki-67-positive cells (Linn *et al*, 1997; Schneider *et al*, 2000). Decrease of Ki-67 expression is not only a monitor of response to treatment, but our data also identify Ki-67 as an independent predictor of response to anthracycline-based chemotherapy. In a comparable study, involving 82 patients from two separate doxorubicin-based neoadjuvant chemotherapy trials, tumours with a high mitotic count had significantly more responses than less proliferating tumours (Wang *et al*, 2002). Probably a proliferating state renders tumour cells more sensitive to chemotherapy: structural damage during DNA synthesis induced by anthracycline exposure decreases the viability of newly formed cancer cells (Collecchi *et al*, 1998; Billgren *et al*, 1999; Makris *et al*, 1999).

In line with reports by others (MacGrogan *et al*, 1996; Kuerer *et al*, 1999), our data also indicate that ER status is a marker of chemosensitivity in breast cancer. Apparently – although functionally unexplained – ER-negative breast carcinomas are more sensitive to anthracycline-based chemotherapy.

With respect to HER2, a recent review of the published data on both adjuvant and neoadjuvant chemotherapy concludes that there is insufficient evidence to use HER2 expression as a predictive factor in breast cancer, as many of the reviewed studies present conflicting data (Hamilton and Piccart, 2000). Our results also reject HER2 status as a predictive marker of response to epirubicin treatment. Studies comparable to ours – comprising a total of 429 patients, in whom the response of the primary tumour to treatment with anthracyclines was measured – have reached the same conclusions (MacGrogan *et al*, 1996; Niskanen *et al*, 1997; Vargas-Roig *et al*, 1999; Vincent-Salomon *et al*, 2000). In contrast, Colleoni *et al* (1999) report that, in 40 patients, HER2-positive tumours (*n*=5) had a significantly increased response rate to neoadjuvant treatment with doxorubicin.

The observed correlation between elevated P53 protein levels and increased chemosensitivity was unexpected, although reported in one earlier publication (Colleoni *et al*, 1999). It opposes the generally accepted view that dysfunctional P53 causes resistance to anthracyclines, as the result of a lacking DNA integrity check (Lowe *et al*, 1993).

The relatively high dose of epirubicin (120 mg m^−2^) employed in these patients may have an additional effect to a lower – and more widely used – dosage of 60–90 mg m^−2^. Possibly, the DNA damage that is initially tolerated with lacking P53 causes decreased viability during subsequent tumour proliferation. In contrast, intact P53 induces apoptosis, or the DNA repair mechanism rescues cancer cell viability.

It may also be that specific mutations necessary to induce anthracycline resistance, described by Aas (Aas *et al*, 1996), were relatively under-represented in our population. Alternatively, a correlation between elevated P53 levels and high proliferation index, as was previously reported (Allred *et al*, 1993), would have explained the apparent sensitivity of P53 positive tumours in our series. We did however find no such association.

Another unexpected finding was that response to chemotherapy was not predictive of outcome. Neither the clinical or pathological, nor the additional integrated response score was predictive of outcome. The response of a tumour is often used as a measure of its chemosensitivity. If the response reflects the chemosensitivity of all tumour cells – disseminated or locoregional – it would be expected that response be positively correlated with outcome. Results of the NSABP trial B-18, evaluating the merits of neoadjuvant chemotherapy for the treatment of stage I and II breast cancer, support this.

In our patients no clinical evidence of distant dissemination was found preoperatively. It is however likely that in a relatively large percentage of this selected population, which had extensive lymph node involvement, subclinical metastases were present. It may be that patients' OS and DFS were not so much determined by the extent of cell kill induced by chemotherapy. Alternatively, the remaining disseminated and chemoresistant tumour cells, irrespective of their numbers, may have been the most important determinant of outcome. In agreement with this explanation is the recently published study by Gajdos *et al* presenting a retrospective analysis of 144 locally advanced breast cancer patients who had been treated with neoadjuvant chemotherapy. The authors found no association between the clinical and pathological response and outcome. However, distant DFS and OS were significantly related to the number of tumour-positive axillary lymph nodes, hinting at a role for tumour cell dissemination independent of primary tumour sensitivity to chemotherapy (Gajdos *et al*, 2002).

The results of this study underline the inadequacy of palpated tumour size as a parameter of breast cancer regression upon cytotoxic treatment. We further show that exposure to anthracycline-based chemotherapy does not result in significant changes of the expression of several possibly predictive markers. Only the rate of proliferation decreased markedly. For a clinical model predicting breast cancer response to anthracycline-based chemotherapy, ER status and Ki-67 count are probably the most useful of the tested markers. However, in this study, response was not associated with outcome, nor was expression of any of the molecular markers.
